# GLP-1 receptor agonist and risk of erectile dysfunction in men with type 2 diabetes: a target trial emulation

**DOI:** 10.1016/j.eclinm.2026.103857

**Published:** 2026-04-02

**Authors:** Huilin Tang, Yiwen Lu, Bingyu Zhang, Dazheng Zhang, David A. Asch, Yong Chen

**Affiliations:** a The Center for Health AI and Synthesis of Evidence (CHASE), University of Pennsylvania, Philadelphia, PA, USA; b Department of Biostatistics, Epidemiology, and Informatics, University of Pennsylvania Perelman School of Medicine, Philadelphia, PA, USA; c The Graduate Group in Applied Mathematics and Computational Science, School of Arts and Sciences, University of Pennsylvania, Philadelphia, PA, USA; d Leonard Davis Institute of Health Economics, University of Pennsylvania, Philadelphia, PA, USA; e Wharton School, University of Pennsylvania, Philadelphia, PA, USA; f Division of General Internal Medicine, Department of Medicine, Perelman School of Medicine, University of Pennsylvania, Philadelphia, PA, USA; g Penn Medicine Center for Evidence Based Practice (CEP), University of Pennsylvania, Philadelphia, PA, USA; h Penn Institute for Biomedical Informatics (IBI), University of Pennsylvania, Philadelphia, PA, USA

**Keywords:** GLP-1RAs, DPP4is, Type 2 diabetes, Erectile dysfunction, Target trial emulation

## Abstract

**Background:**

The association between glucagon-like peptide-1 receptor agonists (GLP-1RAs) and the risk of erectile dysfunction (ED) in men with type 2 diabetes (T2D) remains unclear. This study aimed to evaluate the risk of ED associated with GLP-1RA initiation compared with dipeptidyl peptidase-4 inhibitors (DPP4is) in men with T2D.

**Methods:**

We conducted a target trial emulation using electronic health records from a U.S. health system between January 2019 and September 2024. Adult men (>18 years) with T2D initiating either GLP-1RA or DPP4i were included. The primary outcome was incident ED identified using diagnostic codes. Baseline characteristics were balanced using stabilized inverse probability of treatment weighting (sIPTW), and hazard ratios (HRs) were estimated using Cox proportional hazards models. Multiple subgroup analyses, sensitivity analyses (including negative control outcome [NCO] calibration), and external validation were conducted to assess robustness.

**Findings:**

After sIPTW, this study included 4910 GLP-1RA initiators and 5524 DPP4i initiators with well-balanced baseline covariates. The incidence rate of ED was higher in the GLP-1RA users (35.2 vs. 28.0 per 1000 person-years) than DPP4i users, with a slightly increased rate (HR, 1.26; 95% CI, 1.08–1.46). Results were generally consistent across sensitivity analyses, subgroups, and an external validation cohort, while the association was attenuated and no longer statistically significant after NCO calibration.

**Interpretation:**

In men with T2D, GLP-1RA use was modestly associated with an increased rate of ED. These observational findings may reflect residual or selection bias and do not establish causation. Further studies are warranted to confirm these findings and explore potential underlying mechanisms.

**Funding:**

This work was supported in part by 10.13039/100000002National Institutes of Health, United States (RF1AG077820, R01AG073435, R01DK128237).

**Research in context**

**Evidence before this study**

We searched PubMed on September 3, 2025, for observational studies examining the association between glucagon-like peptide-1 receptor agonists (GLP-1RAs) and erectile dysfunction (ED). The search identified very limited and inconsistent evidence regarding the impact of GLP-1RAs on sexual health outcomes in men with type 2 diabetes (T2D).

**Added value of this study**

Using a large-scale target trial emulation with electronic health record data, we found that initiation of GLP-1RAs was associated with a modestly increased risk of ED compared with initiation of dipeptidyl peptidase-4 inhibitors (DPP4is). This association was robust across sensitivity analyses and was externally validated in the TriNetX database.

**Implications of all the available evidence**

These findings suggest that sexual health outcomes warrant attention in men with T2D receiving GLP-1RAs. While these agents provide well-established metabolic and cardiovascular benefits, clinicians may need to weigh potential risks related to sexual function. Further mechanistic and clinical research is needed to clarify biological pathways.

## Introduction

Erectile dysfunction (ED) is a prevalent complication among men with type 2 diabetes (T2D), affecting nearly 50% of individuals over their lifetime.^1^^,^^2^ The pathophysiology of ED in T2D is multifactorial, primarily attributed to endothelial dysfunction,^3^ impaired nitric oxide signaling,^4^ and autonomic neuropathy,^5^ which collectively contribute to vascular and neurogenic deficits.^6^

Recent studies present conflicting results about the effect of glucagon-like peptide-1 receptor agonists (GLP-1RAs) on sexual function. Preclinical studies suggest that GLP-1RAs may enhance endothelial function, improve oxidative stress, and reduce inflammation, potentially improving erectile function.^7^^,^^8^ An exploratory analysis of a randomized controlled trial (RCT) further indicated that long-term use of dulaglutide may reduce the rate of ED in patients with T2D compared to a placebo (hazard ratio [HR] 0.92, 95% CI 0.85–0.99).^9^ Conversely, some studies indicate that GLP-1RAs may lead to autonomic dysregulation,^10^ hormonal alterations, or adverse effects on testosterone levels, which could contribute to ED.^11^ A population-based cohort study found an increased risk of ED and/or prescription of phosphodiesterase type 5 inhibitors (risk ratio [RR] 4.5, 95% CI 2.3–9.0) and testosterone deficiency (RR 1.9, 95% CI 1.2–3.1) among semaglutide users compared to non-semaglutide users in non-diabetic obese patients.^11^ These contrasting findings reveal the complexity of GLP-1RA effects on sexual health and the need for further investigation.

We conducted a target trial emulation using electronic health records (EHR) from a large multihospital health system to compare the incidence of ED among new users of GLP-1RA with those initiating dipeptidyl peptidase-4 inhibitors (DPP4i). We validated the analysis using a large federated clinical database.

## Methods

### Study design and data sources

We conducted a target trial emulation study using electronic health records (EHRs) from the University of Pennsylvania Health System (Penn Medicine) between January 2019 and September 2024. Our study followed the target trial emulation framework, incorporating eligibility criteria, treatment strategies, assignment procedures, follow-up period, outcome, causal contrasts, and analysis plan aligned with an ideal randomized controlled trial, which we then emulated using observational data (Table S1).^12^ The study was approved by the University of Pennsylvania Institutional Review Board (IRB #853466) and patient informed consent was waived because de-identified data were used. This study was conducted according to the Strengthening the Reporting of Observational Studies in Epidemiology (STROBE) reporting guideline for observational research.^13^

The primary data source was Penn Medicine's EHR repository, which consistents of multiple real-world clinical sites, including the Hospital of the University of Pennsylvania (HUP), Penn Presbyterian Medical Center (PPMC), Pennsylvania Hospital (PAH), Chester County Hospital (CCH), and Penn Medicine Princeton Medical Center (PMC), as well as multiple outpatient practices and affiliated healthcare networks. This dataset encompasses over 6.5 million unique patients and more than 50 million clinical encounters across the Greater Philadelphia metropolitan area, Central Pennsylvania, Delaware, and Southern New Jersey. To validate our findings, we leveraged additional real-world EHR data from the TriNetX network, a global federated research platform that aggregates de-identified patient data from multiple healthcare organizations.^14^ TriNetX provides extensive information on patient demographics, diagnoses, medications, laboratory results, procedures, and clinical outcomes, making it a robust resource for assessing the real-world effectiveness and safety of medical interventions.

### Study population

Adult men aged >18 years with a diagnosis of T2D who initiated treatment with a GLP-1RA or DPP4i were included. Patients with T2D were identified using International Classification of Diseases, Tenth Revision (ICD-10) diagnostic codes (E11). Patients were required to have at least one medical visit in the EHR system within 1 year before treatment initiation to ensure adequate baseline assessment. Exclusion criteria included a prior diagnosis of ED or end-stage renal disease/dialysis before drug initiation to reduce confounding.

### Exposure and comparator definition

Patients were categorized into two cohorts based on the glucose-lowering drug initiated at cohort entry: GLP-1RA or DPP4i (Table S2). Drug initiation (index date) was defined as the first recorded prescription for a medication within a given drug class, with no prior use of that class in the preceding 1 year, a washout period commonly used in target trial emulation to ensure inclusion of new users and minimize potential biases related to prior exposure.^15^, ^16^, ^17^ DPP4is were selected as active comparators because they were recommended as second-line glucose-lowering therapies in clinical guidelines during the study period^18^ and were commonly used as comparators in studies evaluating the safety of GLP-1RAs.^17^^,^^19^

### Outcome assessment and follow-up

The primary outcome was the incidence of ED, identified using ICD-10 diagnostic codes (N52) recorded in the EHR system. An incident case of ED was defined as the first occurrence of an ED diagnosis documented after the initiation of the study medication. The ICD-10 codes are shown in Table S3.

The patients were followed from the date of drug initiation until the earliest occurrence of ED diagnosis, death, or end of study period (September 30, 2024), following an intent-to-treat (ITT) approach, in which patients remained in their originally assigned treatment group regardless of any subsequent changes in therapy.^20^ This approach aims to preserve comparability between groups and reflects real-world treatment effectiveness.^21^

### Baseline covariates

To control for potential confounding, we obtained a comprehensive set of baseline covariates from EHRs, selected based on prior studies and clinical relevance (as shown in Table 1).^22^^,^^23^ These included demographic characteristics such as age, sex, and race/ethnicity; clinical parameters including baseline glycated hemoglobin (HbA1c) levels, body mass index (BMI), and blood pressure (BP); and a range of comorbid conditions (identified using ICD-10 diagnosis codes), such as diabetes-related complications (e.g., neuropathy, retinopathy), hypertension, obesity, coronary heart disease (CHD), and chronic kidney disease (CKD). Also included was the use of other glucose lowering therapies (e.g., insulin and metformin), antihypertensive medications, and lipid-lowering agents, which may influence both treatment selection and outcomes. Missing laboratory and vital sign data were handled using single imputation, with all baseline covariates included in the imputation model.^24^

**Table 1:** Baseline characteristics of patients between GLP-1RA and. DPP4i groups using Penn Medicine EHR data.^a^

	Before sIPTW			After sIPTW		
	GLP-1RA (n = 4864)	DPP4i (n = 5568)	SMD	GLP-1RA (n = 4910)	DPP4i (n = 5524)	SMD
Mean age, yrs	58.74 (12.54)	66.18 (12.99)	0.583	62.54 (12.42)	62.75 (13.71)	0.016
Race/ethnicity						
Hispanic	245 (5.0)	318 (5.7)	0.266	260 (5.3)	315 (5.7)	0.022
Non-Hispanic White	2651 (54.5)	3136 (56.3)		2717 (55.3)	3066 (55.5)	
Non-Hispanic Black	1369 (28.1)	1043 (18.7)		1118 (22.8)	1253 (22.7)	
Other/unknown	599 (12.3)	1071 (19.2)		815 (16.6)	890 (16.1)	
Health insurance						
Commercial	2235 (45.9)	1724 (31.0)	0.417	1845 (37.6)	2080 (37.7)	0.014
Medicare	1543 (31.7)	2677 (48.1)		1982 (40.4)	2234 (40.4)	
Medicaid	542 (11.1)	336 (6.0)		414 (8.4)	480 (8.7)	
Other/unknown	544 (11.2)	831 (14.9)		669 (13.6)	730 (13.2)	
Enrollment year			0.356			0.032
2019	1126 (23.1)	1783 (32.0)		1397 (28.5)	1595 (28.9)	
2020	687 (14.1)	1067 (19.2)		806 (16.4)	918 (16.6)	
2021	487 (10.0)	620 (11.1)		535 (10.9)	607 (11.0)	
2022	807 (16.6)	897 (16.1)		810 (16.5)	929 (16.8)	
2023	1079 (22.2)	662 (11.9)		807 (16.4)	844 (15.3)	
2024	678 (13.9)	539 (9.7)		555 (11.3)	632 (11.4)	
Charlson Comorbidity Index			0.383			0.036
≤1	1066 (21.9)	1971 (35.4)		1471 (30.0)	1593 (28.8)	
2–5	2212 (45.5)	1601 (28.8)		1786 (36.4)	1980 (35.8)	
≥6	1586 (32.6)	1996 (35.8)		1653 (33.7)	1951 (35.3)	
Emergency visits (≥1)	509 (10.5)	493 (8.9)	0.055	471 (9.6)	559 (10.1)	0.018
Inpatient visits (≥1)	683 (14.0)	1247 (22.4)	0.218	946 (19.3)	1048 (19.0)	0.008
Outpatient visits						
0	382 (7.9)	731 (13.1)	0.256	544 (11.1)	595 (10.8)	0.01
1–20	2090 (43.0)	2733 (49.1)		2328 (47.4)	2617 (47.4)	
>20	2392 (49.2)	2104 (37.8)		2039 (41.5)	2312 (41.8)	
**Commorbidites**						
Diabtetic nephropathy	509 (10.5)	797 (14.3)	0.117	640 (13.0)	730 (13.2)	0.005
Diabetic retinopathy	208 (4.3)	167 (3.0)	0.068	172 (3.5)	236 (4.3)	0.04
Diabetic neuropathy	436 (9.0)	486 (8.7)	0.008	452 (9.2)	520 (9.4)	0.007
Peripheral vascular disease	196 (4.0)	277 (5.0)	0.046	235 (4.8)	244 (4.4)	0.018
Other unspecified diabetic complications	319 (6.6)	261 (4.7)	0.081	272 (5.5)	330 (6.0)	0.019
Acute myocardial infarction	117 (2.4)	168 (3.0)	0.038	143 (2.9)	152 (2.7)	0.01
Coronary artery disease	869 (17.9)	1210 (21.7)	0.097	993 (20.2)	1095 (19.8)	0.01
Arrhythmias	650 (13.4)	1058 (19.0)	0.154	826 (16.8)	909 (16.5)	0.01
Cardiomyopathy	258 (5.3)	366 (6.6)	0.054	315 (6.4)	336 (6.1)	0.014
Hypertension	2967 (61.0)	3152 (56.6)	0.089	2903 (59.1)	3256 (59.0)	0.004
Lipid disorders	2688 (55.3)	2928 (52.6)	0.054	2619 (53.3)	2968 (53.7)	0.008
Cerebrovascular disease	217 (4.5)	439 (7.9)	0.143	341 (6.9)	350 (6.3)	0.024
Alcohol-related disorders	85 (1.7)	125 (2.2)	0.036	120 (2.4)	123 (2.2)	0.014
Anxiety disorders	385 (7.9)	353 (6.3)	0.061	367 (7.5)	395 (7.2)	0.012
Asthma	282 (5.8)	210 (3.8)	0.095	239 (4.9)	277 (5.0)	0.006
COPD	231 (4.7)	346 (6.2)	0.064	289 (5.9)	310 (5.6)	0.012
Bronchitis	69 (1.4)	98 (1.8)	0.027	82 (1.7)	92 (1.7)	<0.001
Pneumonia	99 (2.0)	220 (4.0)	0.113	161 (3.3)	183 (3.3)	0.001
Chronic kidney disease	511 (10.5)	937 (16.8)	0.185	702 (14.3)	790 (14.3)	0.001
COVID-19	163 (3.4)	161 (2.9)	0.026	143 (2.9)	167 (3.0)	0.006
Thyroid disorders	104 (2.1)	98 (1.8)	0.027	99 (2.0)	110 (2.0)	0.003
Pancreatic disorders	49 (1.0)	88 (1.6)	0.051	59 (1.2)	72 (1.3)	0.01
Biliary tract disease	75 (1.5)	114 (2.0)	0.038	82 (1.7)	100 (1.8)	0.009
Glaucoma	149 (3.1)	147 (2.6)	0.025	142 (2.9)	151 (2.7)	0.01
Cognitive impairment	130 (2.7)	297 (5.3)	0.136	191 (3.9)	232 (4.2)	0.015
Fractures	91 (1.9)	144 (2.6)	0.048	151 (3.1)	123 (2.2)	0.052
Obesity	1809 (37.2)	931 (16.7)	0.474	1280 (26.1)	1424 (25.8)	0.007
Osteoarthritis	477 (9.8)	448 (8.0)	0.062	444 (9.0)	474 (8.6)	0.016
Skin cancers	83 (1.7)	123 (2.2)	0.036	81 (1.6)	102 (1.8)	0.015
Malnutrition	63 (1.3)	209 (3.8)	0.157	160 (3.3)	152 (2.8)	0.03
**Medications**						
ARB/ACEi	2875 (59.1)	3282 (58.9)	0.003	2955 (60.2)	3296 (59.7)	0.011
Calcium-channel blockers	1605 (33.0)	1928 (34.6)	0.034	1702 (34.7)	1918 (34.7)	0.001
Diuretics	2208 (45.4)	2716 (48.8)	0.068	2359 (48.0)	2662 (48.2)	0.003
Lipid-lowering drugs	3370 (69.3)	4012 (72.1)	0.061	3505 (71.4)	3958 (71.7)	0.006
Corticosteroids	770 (15.8)	926 (16.6)	0.022	778 (15.8)	910 (16.5)	0.017
NSAIDs	1124 (23.1)	1134 (20.4)	0.067	1040 (21.2)	1206 (21.8)	0.016
Antidepressants	1040 (21.4)	1058 (19.0)	0.059	1005 (20.5)	1120 (20.3)	0.005
Antipsychotics	319 (6.6)	492 (8.8)	0.086	422 (8.6)	451 (8.2)	0.016
Antidementia drugs	31 (0.6)	110 (2.0)	0.118	78 (1.6)	73 (1.3)	0.022
Anticoagulants	433 (8.9)	703 (12.6)	0.12	565 (11.5)	640 (11.6)	0.002
Antiplatelet agents	1356 (27.9)	2064 (37.1)	0.197	1668 (34.0)	1863 (33.7)	0.006
Proton pump inhibitors	1132 (23.3)	1518 (27.3)	0.092	1275 (26.0)	1388 (25.1)	0.019
Opioids	1209 (24.9)	1740 (31.2)	0.143	1441 (29.3)	1587 (28.7)	0.014
Other anti-obesity medication	247 (5.1)	161 (2.9)	0.112	191 (3.9)	208 (3.8)	0.006
Insulin	1736 (35.7)	2204 (39.6)	0.08	1918 (39.1)	2191 (39.7)	0.012
Metformin	2939 (60.4)	3564 (64.0)	0.074	3087 (62.9)	3480 (63.0)	0.003
Sulfonylureas	822 (16.9)	1575 (28.3)	0.275	1204 (24.5)	1341 (24.3)	0.006
Thiazolidinediones	118 (2.4)	224 (4.0)	0.09	166 (3.4)	189 (3.4)	0.002
a-glucosidase inhibitors	7 (0.1)	26 (0.5)	0.059	15 (0.3)	17 (0.3)	<0.001
SGLT2i	40 (0.8)	29 (0.5)	0.037	31 (0.6)	48 (0.9)	0.027
**Lab and vital values**						
**After imputation**^b^						
HbA1c, %	8.07 (1.91)	8.02 (1.74)	0.028	8.10 (1.85)	8.14 (1.79)	0.022
BMI, kg/m^2^	35.26 (7.26)	30.50 (6.03)	0.713	32.79 (6.83)	32.75 (7.23)	0.005
Systolic blood pressure, mmHg	132.00 (16.18)	131.77 (17.62)	0.014	132.23 (16.85)	132.09 (17.20)	0.008
Diastolic blood pressure, mmHg	78.96 (9.96)	76.35 (10.38)	0.257	77.72 (10.12)	77.67 (10.52)	0.004
**Before imputation**						
Hemoglobin A1c, %	8.19 (1.96)	8.16 (1.73)	0.019	8.24 (1.90)	8.32 (1.80)	0.047
Body mass index, kg/m^2^	35.39 (7.26)	30.47 (6.02)	0.738	32.94 (6.85)	32.78 (7.30)	0.023
Systolic blood pressure, mmHg	131.96 (16.06)	131.74 (17.57)	0.013	132.01 (16.74)	132.10 (17.12)	0.005
Diastolic blood pressure, mmHg	78.87 (9.90)	76.29 (10.35)	0.255	77.65 (10.14)	77.65 (10.51)	0.001

Abbreviations: ACEi, angiotensin-converting enzyme inhibitor; ARB, angiotensin-receptor blocker; BMI, body mass index; COPD, chronic obstructive pulmonary disease; DPP4i, dipeptidyl peptidase-4 inhibitor; GLP-1RA, glucagon-like peptide-1 receptor agonist; NSAID, nonsteroidal anti-inflammatory drug; SGLT2i, sodium-glucose cotransporter-2 inhibitors; SMD, standardized mean difference; sIPTW, stabilized inverse probability treatment weighting.

a Values are numbers (percentages) unless otherwise indicated.

b Missing values were handled using single imputation. The proportions of missing data were 40.3% for Hemoglobin A1c, 11.4% for body mass index, 7.3% for systolic blood pressure, 7.4% for diastolic blood pressure. Only imputed values were adjusted in sIPTW.

### Statistical analysis

To address potential confounding and improve comparability between treatment groups, we used stabilized inverse probability of treatment weighting (sIPTW) based on propensity scores derived from logistic regression models.^25^ The propensity score model incorporated a comprehensive set of baseline covariates, as detailed in Table 1. We evaluated the balance of baseline covariates with standardized mean differences (SMDs) before and after weighting, with an SMD of <0.1 indicating an adequate balance.^26^ To further assess balance, we used love plots to visualize the distribution of covariates before and after weighting. Association between GLP-1RA initiation and the rate of ED, compared with DPP4is, were estimated using Cox proportional hazards regression models, with hazard ratios (HRs) and 95% confidence intervals (CIs) reported before and after applying sIPTW.

We performed subgroup analyses to explore potential effect modification by key patient characteristics, including: 1) Age ((<45 years vs. ≥ 45 years and <65 years vs. ≥65 years); 2) Race and ethnicity (Hispanic vs. non-Hispanic White vs. non-Hispanic Black vs. other); 3) Obesity status (yes vs. no); 4) Presence of coronary heart disease (yes vs. no); 5) Presence of CKD (yes vs. no); 6) Presence of hypertension (yes vs. no); 7) Insulin use at baseline (yes vs. no); 8) Metformin use at baseline (yes vs. no); 9) individual GLP-1RAs (semaglutide vs. tirzepatide vs. liraglutide vs. dulaglutide). To ensure covariate balance within each subgroup, we refitted the sIPTW model separately within each stratum. To assess the robustness of the primary findings, we also conducted several sensitivity analyses, including: 1) using standard IPTW as an alternative weighting method; 2) conducting 1:1 propensity score matching (PSM) using nearest-neighbor matching with a caliper of 0.1 of the pooled standard deviation of the logit of the propensity score; 3) using SGLT2i as an alternative active comparator, another non-insulin glucose-lowering alternatives to GLP-1RAs; 4) applying negative control outcome (NCO) calibration to address potential residual confounding and systematic biases, using a list of 38 NCOs with no plausible causal relationships to exposure (Table S4).^27^^,^^28^ An empirical null distribution was derived from the NCO effect estimates obtained using the same analytic framework as the primary outcome and was used to generate calibrated effect estimates and CIs.

We also performed an external validation using the EHR data from TriNetX U.S. Collaborative Network,^14^ using 1:1 PSM to replicate the primary findings in an independent dataset (details provided in Text S1). Statistical analyses were conducted using R (version 4.5.2), with two-sided p-values <0.05 considered statistically significant.

### Role of the funding source

This study was non-commercially funded by the National Institutes of Health. No pharmaceutical company or external agency was involved in the development of the manuscript. The funders had no role in study design, data collection, data analysis, data interpretation, or writing of the report. The authors had full access to all the data in the study and had final responsibility for the decision to submit for publication.

## Results

### Baseline characteristics

The flowchart of patient selection is presented in Figure S1. Based on the inclusion and exclusion criteria, a total of 4864 GLP-1RA users and 5568 DPP4i users were included in the GLP-1RA vs. DPP4i cohort. Baseline characteristics from Penn Medicine are presented in Table 1. GLP-1RA users were younger (mean [SD] age: 58.7 [12.5] vs. 66.2 [13.0] years) and had a higher prevalence of obesity (37.2% vs. 16.7%) compared to DPP4i users. Laboratory values also differed, with higher BMI (35.3 vs. 30.5 kg/m^2^) and eGFR (86.1 vs. 75.9 mL/min/1.73 m^2^) observed in the GLP-1RA group. After applying sIPTW, baseline imbalances were substantially reduced, with SMDs below 0.1 for key covariates, enhancing the validity of subsequent comparative analyses (Table 1 and loveplot shown in Figure S2). In the TriNetX dataset, among 224,022 GLP-1RA initiators and 114,660 DPP4i initiators (Table S5), 98,131 matched pairs were identified after applying 1:1 PSM, and baseline characteristics were well balanced post-matching (Table S6).

### Primary analysis

**Fig. 1:**
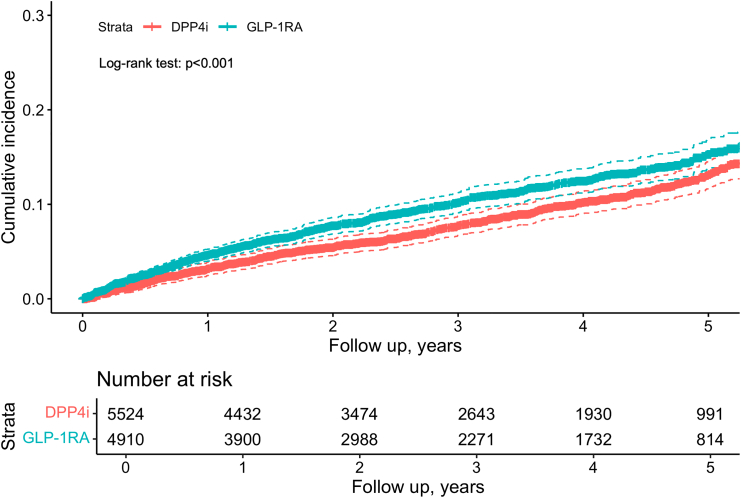
Stabilized inverse probability of treatment weight (sIPTW) adjusted cumulative incidence of erectile dysfunction with GLP-1RA compared to DPP4i using Penn Medicine electronic health record data. The solid line represents the estimated cumulative incidence, and the dashed lines indicate the 95% confidence intervals. GLP-1RA, glucagon-like peptide-1 receptor agonist; DPP4i, dipeptidyl peptidase-4 inhibitor.

The results from the primary analysis are presented in Table 2. In the cohort comparing GLP-1RA users (n = 4864) with DPP4i users (n = 5568), the mean follow-up duration was 2.58 years (SD: 1.77) for GLP-1RA users and 3.08 years (SD: 1.80) for DPP4i users. Before sIPTW adjustment, the incidence rate (IR) of ED was higher in the GLP-1RA group (44.4 per 1000 person-years) compared to the DPP4i group (24.6 per 1000 person-years), with an HR of 1.78 (95% CI: 1.57–2.02) and a p-value of <0.001. After applying sIPTW, the IR remained higher among GLP-1RA users (35.2 vs. 28.0 per 1000 person-years), while the HR decreased to 1.26 (95% CI: 1.08–1.46) with a p-value of 0.004, compared with the unadjusted estimate. This indicates that baseline confounders likely accounted for a substantial portion of the initially observed increased rate. The sIPTW adjusted Kaplan–Meier curve showing the cumulative incidence of ED between the two groups is shown in Fig. 1.

**Table 2:** The association between GLP-1 RA and rate of erectile dysfunction compared to DPP4i using Penn Medicine EHR data.

	GLP-1RA	DPP4i
**Before applying sIPTW**		
Events/Patients at risk, n/N	557/4864	422/5568
Mean follow-up (sd), yrs	2.58 (1.77)	3.08 (1.80)
IR, per 1000 person-years	44.4	24.6
HR (95% CI)	1.78 (1.57, 2.02)	Reference
p-value	<0.001	Reference
**After applying sIPTW**		
Events/Patients at risk, n/N	495/4910	451/5524
Mean follow-up (sd), yrs	2.86 (1.81)	2.92 (1.80)
IR, per 1000 person-years	35.2	28.0
HR (95% CI)	1.26 (1.08, 1.46)	Reference
p-value	0.004	Reference

Abbreviations: sIPTW, stabilized inverse probability of treatment weight; GLP-1RA: glucagon-like peptide-1 receptor agonist; DPP4i, dipeptidyl peptidase-4 inhibitor; HR, hazard ratio; IR, incidence rate; CI, confidence interval.

### Subgroup and sensitivity analyses

**Fig. 2:**
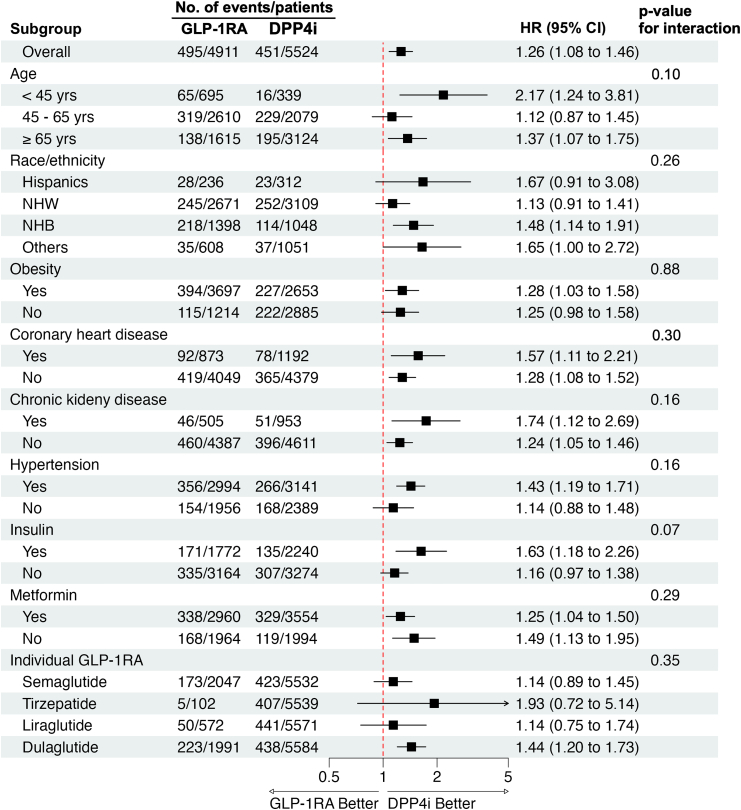
Subgroup analyses of the association between GLP-1RA use and rate of erectile dysfunction compared to DPP4i use using Penn Medicine electronic health record data. GLP-1RA: glucagon-like peptide-1 receptor agonist; DPP4i, dipeptidyl peptidase-4 inhibitor. NHW, non-Hispanic White; NHB, non-Hispanic Blacks; HR, hazard ratio; CI, confidence interval.

**Fig. 3:**
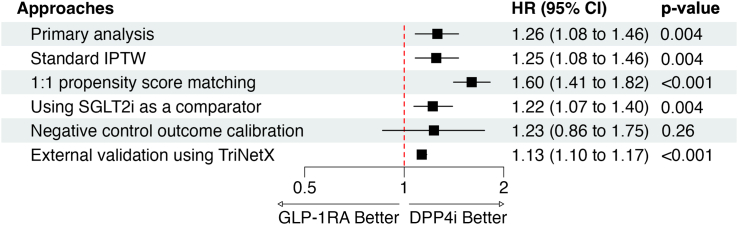
Sensitivity analyses and external validation of the association between GLP-1RA use and rate of erectile dysfunction compared to DPP4i use. GLP-1RA, glucagon-like peptide-1 receptor agonist; DPP4i, dipeptidyl peptidase-4 inhibitor; IPTW, inverse probability of treatment weight; HR, hazard ratio; CI, confidence interval.

Subgroup analyses showed that the association between GLP-1RA use and increased rate of ED was generally consistent across demographic and clinical subgroups (Fig. 2). Analyses by individual GLP-1RA agents suggested a higher rate with dulaglutide, while estimates for other agents were modest or imprecise due to small sample sizes, and no significant interaction was observed across subgroups. Further sensitivity analyses using 1:1 PSM, standard IPTW, and SGLT2i as an alternative comparator confirmed the robustness of these findings (Fig. 3). Although the overall trend remained similar after NCO calibration, the association was attenuated and not statistically significant. The empirical null distribution of NCOs, with their HRs and corresponding standard errors, is presented in Figure S3. The validation analysis using TriNetX data yielded similar results, with an HR of 1.13 (95% CI, 1.10–1.17) and a p-value of <0.001, over a mean follow-up of 2.68 years (SD: 1.62) for the GLP-1RA group and 3.31 s years (SD: 1.99) for the DPP4i group (Fig. 3).

## Discussion

In this target trial emulation, initiation of GLP-1RAs was associated with a higher rate of ED among men with T2D compared with initiation of DPP4is. This association was observed consistently across multiple sensitivity analyses, including 1:1 PSM, and was generally robust across clinically relevant subgroups. Directionally similar findings were also observed in an external validation cohort, although the magnitude of the association was attenuated, suggesting potential differences in population characteristics, outcome ascertainment, or residual confounding across data sources. Importantly, the association was further attenuated and not statistically significant after NCO calibration, indicating that unmeasured confounding or systematic biases may have influenced the primary estimates. In addition, ED was identified using administrative diagnosis codes, which may undercapture milder cases and introduce misclassification. Together, these observational findings should be interpreted cautiously, as they do not establish causation and may be affected by residual or selection bias.

These findings align with a recent cohort study using the TriNetX database, which reported a higher risk of ED and increased use of phosphodiesterase type 5 inhibitors among semaglutide users compared to non-users in non-diabetic patients with obesity.^11^ Together, these results raise questions about the potential impact of GLP-1RAs on male sexual health across diverse populations. The biological mechanisms underlying this association remain incompletely understood. One plausible hypothesis is that GLP-1RAs may influence autonomic nervous system function,^29^ which is essential for erectile response. Preclinical studies have suggested that GLP-1RAs may alter sympathetic-parasympathetic balance,^30^^,^^31^ possibly affecting vascular tone and penile blood flow. Additionally, emerging evidence suggests that GLP-1RAs may modulate sex hormone levels,^11^^,^^32^ which could further impair erectile function.

However, the relationship between GLP-1RA use and ED is complex. Some preclinical and early clinical studies have reported potentially beneficial effects, including improvements in endothelial function,^33^ reductions in oxidative stress,^7^ and decreased systemic inflammation,^8^ all of which may support erectile health. Moreover, an exploratory analysis from the REWIND trial suggested a modest reduction in rate of ED with dulaglutide.^9^ These conflicting findings between real-world evidence and trial-based data underscore the need for further mechanistic and prospective studies to clarify the net effect of GLP-1RAs on sexual health.

This study has several notable strengths. First, the target trial emulation design improves causal inference by closely approximating a randomized controlled trial within a real-world dataset. Second, sIPTW effectively balanced baseline covariates, and NCO calibration addressed potential unmeasured confounding. Third, replication in an external validation cohort enhanced the robustness and generalizability of the findings.

However, several limitations should be considered when interpreting these findings. First, although extensive adjustment was performed using sIPTW, residual confounding cannot be completely excluded. Important clinical factors such as diabetes duration and detailed pre-baseline medication history were not consistently available, despite their relevance to ED risk in long-standing and treatment-resistant diabetes.^34^ To partially address this, our models adjusted for downstream indicators of disease severity, including insulin use and diabetes-related complications such as neuropathy and retinopathy,^35^^,^^36^ which were defined using ICD-10 diagnosis codes. In addition, NCO calibration was applied to further reduce the impact of systematic/residual bias. Second, users of GLP-1RAs are more likely to be overweight or obese, and adiposity itself is a well-established risk factor for ED.^37^ Although baseline obesity status and BMI were explicitly adjusted for in the weighting models and subgroup analyses demonstrated generally consistent results, residual confounding and selection bias related to metabolic health cannot be fully excluded. Moreover, post-initiation changes in body weight and glycemic control, plausible mediators of the association between GLP-1RA use and ED, were not examined. Because the primary estimand was the total effect of treatment initiation, adjustment for these post-baseline variables could introduce bias; however, the inability to formally assess mediation limits mechanistic interpretation. Third, exposure was defined using a new-user, ITT–like framework, whereby individuals were classified at treatment initiation and followed regardless of subsequent adherence, discontinuation, or switching.^21^ While this approach reflects real-world treatment patterns and preserves baseline covariate balance, it precludes evaluation of treatment persistence, duration, cumulative exposure, and dose–response relationships. As-treated or per-protocol analyses may better capture the effects of sustained exposure, but such approach may introduce selection bias and time-varying confounding, potentially compromising baseline comparability and generalizability.^38^ In addition, reliance on prescription records available in EHR data limited our ability to perform such analyses. Fourth, ED outcomes were identified using ICD-10 diagnosis codes recorded in EHRs. ED is frequently underreporting or underdiagnosed and variably documented across healthcare organizations, and diagnostic criteria may differ between providers. As a result, outcome misclassification and measurement bias are possible, which may have influenced effect estimates. Fifth, missing data in laboratory and vital sign measurements represent an additional limitation. HbA1c, BMI, and blood pressure were imputed using single imputation to maintain computational feasibility within the target trial emulation. While single imputation does not fully account for imputation uncertainty and may underestimate variance,^24^ mean and standard deviation values were largely similar before and after imputation. Finally, this study included men with T2D in a healthcare system, with external validation using the TriNetX U.S. Collaborative Network, which may limit generalizability to non-U.S. populations or individuals without T2D (e.g., those with obesity). Overall, these limitations suggest that the results should be interpreted cautiously and viewed as hypothesis-generating. Future randomized controlled trials with standardized, validated assessment of ED in all participants and longitudinal measurement of treatment exposure, metabolic changes, and adherence are needed to minimize residual confounding and measurement bias and to clarify the causal relationship between GLP-1RA use and sexual health outcomes.

In conclusion, our findings suggest a potential association between GLP-1RA use and an increased rate of ED in adults with T2D, contrasting with results from a post-hoc trial analysis. These observational results are hypothesis-generating, do not establish causation, and may reflect residual or selectin bias. Future randomized controlled trials with standardized assessment of erectile function and comprehensive longitudinal measurement of treatment exposure are needed to confirm these findings and elucidate the underlying mechanisms.

## Contributors

HT and YC conceptualized and designed the study, and YC supervised the project. HT and BZ conducted the statistical analyses. HT, YL, BZ, DZ, DAA, and YC contributed to data interpretation. YC secured funding for the study. HT drafted the initial manuscript. HT, YL, BZ, DZ, DAA, and YC critically reviewed the manuscript and approved the final version. HT, YL, and BZ had full access to and verified the underlying data reported in the manuscript.

## Data sharing statement

The data used in this study from Penn Medicine and the TriNetX research network are not publicly available due to patient privacy and institutional policies. Access to data from the TriNetX network may be requested directly from TriNetX (www.trinetx.com), subject to their data use agreements and approval processes.

## Declaration of interests

All authors declare no conflict of interest.
