# Supplementary material for: GLP-1 receptor agonist and risk of erectile dysfunction in men with type 2 diabetes: a target trial emulation
Source: eClinicalMedicine. 2026 Apr 2;94:103857. doi: 10.1016/j.eclinm.2026.103857 (PMC13084313; doi:10.1016/j.eclinm.2026.103857)
Supplement: Supplement [file mmc1.docx]

**Supplementary material**

**Contents**

**Supplement Table 1**. Target trials emulation.

**Supplement Table 2**. Exposures of interest and comparison.

**Supplement Table 3.** Codes used for outcome definition and inclusion and exclusion criteria.

**Supplement Table 4.** List of negative control outcomes.

**Supplement Table 5.** The selection of the population cohort.

**Supplement Table 6.** Baseline characteristics of patients between GLP-1RA and. DPP4i groups using electronic health record data from TriNetX U.S. Collaborative Network (Jan 1, 2019 to Sep 30, 2024)

**Supplement text 1.** External Validation Using TriNetX Research Network

**Supplement Figure 1.** The flowchart of patient selection using Penn Medicine electronic health record (EHR) data.

**Supplement Figure 2.** Love plot of the absolute standardized mean difference (SMD) for baseline covariates before and after stabilized inverse probability of treatment weighting (sIPTW)

**Supplement Figure 3.** Empirical null distribution of negative control outcomes (NCOs).

**Supplement Table 1**. Target trials emulation

| Approaches | Target trial | Target Trial Emulation |
| --- | --- | --- |
| Eligibility criteria | - Men - Type 2 diabetes - Age >18 years - No prescription for GLP-1RA or DPP4i within past year - No prior diagnosis of type 1 diabetes or erectile dysfunction - No contraindication to study drugs (end-stage renal disease/dialysis) | Same as for the target trial except that we required at least 1 medical visit in the electronic health record system within one year before treatment initiation |
| Treatment strategies | Initiation treatment with a GLP-1RA or DPP4i | The date of initiation treatment was the date of the first prescription of a GLP-1RA or DPP4i |
| Treatment assignment | Individuals are randomly assigned to GLP-1RA or DPP4i at baseline. Individuals and their treating physicians will be aware of the assigned treatment strategy | Treatment is not assigned randomly.  Randomization was emulated using stabilized inverse probability of treatment weighting (sIPTW) to address potential confounders. |
| Outcomes | Incident erectile dysfunction is identified using diagnosis codes. | Same as for the target trial |
| Follow-up | For each eligible individual, follow-up starts at treatment assignment and ends on the onset of erectile dysfunction, death, or the end of the study (30 September 2024), whichever comes first | Same as for the target trial. |
| Causal contrasts | Intention-to-treat effect | Observational analogs of the intention-to-treat |
| Statistical analysis | Kaplan–Meier survival analysis of erectile dysfunction is used to estimate the survival curves and Cox proportional hazard regression models are used to estimate hazard ratios with 95% CIs.  Subgroup analyses by 1) Age (<65 years vs. ≥65 years); 2) Race and ethnicity (Hispanic vs. non-Hispanic White vs. non-Hispanic Black vs. other); 3)Obesity status (yes vs. no); 4) Presence of coronary hear disease (yes vs. no); 5) Presence of chronic kidney disease (yes vs. no); 6) Presence of hypertension (yes vs. no); 7) Insulin use at baseline (yes vs. no); 8) Metformin use at baseline (yes vs. no). | Same as for the target trial  Same subgroup analyses.  Sensitivity analyses: 1)standard IPTW; 2) 1:1 propensity score matching; 3)Negative control outcome (NCO) calibration. |

GLP-1RAs, glucagon-like peptide-1 receptor agonists; DPP4i, dipeptidyl peptidase 4 inhibitors; SGLT2 Inhibitors, sodium-glucose cotransporter 2 inhibitors; GLD, glucose-lowering drug, CI, confidence interval.

**Supplement Table 2**. Exposures of interest and comparison

| **Category** | **Medications** |
| --- | --- |
| GLP-1RAs | Dulaglutide, Exenatide, Liraglutide, Lixisenatide, Semaglutide, Tirzepatide |
| DPP4i | Sitagliptin, Saxagliptin, Alogliptin, Linagliptin |

GLP-1RAs, glucagon-like peptide-1 receptor agonists; DPP4i, dipeptidyl peptidase 4 inhibitors.

**Supplement Table 3.** Codes used for outcome definition and inclusion, and exclusion criteria

| **Outcome/Inclusion and exclusion criteria** | **ICD-10** |
| --- | --- |
| Type 2 diabetes | E11 |
| Type 1 diabetes | E10 |
| Erectile dysfunction | N52 |
| End-Stage Renal Disease /dialysis | N18.5, N18.6, R88.0, T82.4, T85.611, T85.621, T85.631, T85.71, T86.1, Y84.1, Z48.22, Z49, Z94.0, Z99.2 |

**Supplement Table 4.** List of 38 negative control outcomes

**Outcomes**

Abnormal Posture

Abnormal Pupil

Abrasion and Friction Burn of Multiple Sites

Abrasion and Friction Burn of Trunk Without Infection

Absent Kidney

Acquired Keratoderma

Benign Paroxysmal Positional Vertigo

Calcaneal Spur

Chondromalacia of Patella

Contact Dermatitis

Contusion of Knee

Derangement of Knee

Deviated Nasal Septum

Epidermoid Cyst

Feces Contents Abnormal

Foreign Body in Ear

Foreign Body in Orifice

Ganglion Cyst

Genetic Disorder Carrier

Hammer Toe

Homocystinuria

Impacted Cerumen

Impingement Syndrome of Shoulder Region

Ingrowing Nail

Lagophthalmos

Lipid Storage Disease

Lymphangioma

Macular Drusen

Mechanical Complication of Internal Orthopedic Device Implant Graft

Noise Effects on Inner Ear

Nonspecific Tuberculin Test Reaction

Presbyopia

Regular Astigmatism

Senile Hyperkeratosis

Sprain of Ankle

Tear Film Insufficiency

Tooth Loss

**Supplement Table 5.** **The selection of the population cohort.** HCOs, healthcare organizations. GLP-1RA, glucagon-like peptide-1 receptor agonists; DPP4i, dipeptidyl peptidase-4 inhibitors.

| **GLP-1RA cohort** | **Patients** | **HCOs** |
| --- | --- | --- |
| **Base Population** | 128,830,664 | 72 |
| **Population Any age, Male** | 59,916,903 | 72 |
| **Group 1A -** The terms in this group occurred between Jan 01, 2019 and Sep 30, 2024. **Must Have:** ATC A10BJ Glucagon-like peptide-1 (GLP-1) analogues [≥ 18 years] OR RxNorm 2601723 tirzepatide [≥ 18 years]  **Group 1B -** Any instance of Group 1B occurred on or before the first instance of Group 1A. **Must Have:** ICD-10-CM E11 Type 2 diabetes mellitus **Cannot Have:** ICD-10-CM N18.6 End stage renal disease ICD-10-CM Z99.2 Dependence on renal dialysis ICD-10-CM E10 Type 1 diabetes mellitus ATC A10BH Dipeptidyl peptidase 4 (DPP-4) inhibitors ICD-10-CM N52 Male erectile dysfunction | 265,419 | 71 |
| **Group 2A -** The terms in this group occurred between Jan 01, 2019 and Sep 30, 2024. **Must Have:** ATC A10BJ Glucagon-like peptide-1 (GLP-1) analogues [≥ 18 years] OR RxNorm 2601723 tirzepatide [≥ 18 years]  **Group 2B -** Any instance of Group 2B occurred within 1 day and 1 year before the first instance of Group 2A. **Must Have:** Visit **Cannot Have:** ATC A10BJ Glucagon-like peptide-1 (GLP-1) analogues RxNorm 2601723 tirzepatide | 225,242 | 71 |
| Results did not return | 1,220 | 71 |
|  | **224,022** | **71** |
|  | Patients | HCOs |

| **DPP4i cohort** | **Patients** | **HCOs** |
| --- | --- | --- |
| **Base Population** | 128,830,664 | 72 |
| **Population Any age, Male** | 59,916,903 | 72 |
| **Group 1A -** The terms in this group occurred between Jan 01, 2019 and Sep 30, 2024. **Must Have:** ATC A10BH Dipeptidyl peptidase 4 (DPP-4) inhibitors [≥ 18 years]  **Group 1B -** Any instance of Group 1B occurred on or before the first instance of Group 1A. **Must Have:** ICD-10-CM E11 Type 2 diabetes mellitus **Cannot Have:** ICD-10-CM N18.6 End stage renal disease ICD-10-CM Z99.2 Dependence on renal dialysis ATC A10BJ Glucagon-like peptide-1 (GLP-1) analogues RxNorm 2601723 tirzepatide ICD-10-CM E10 Type 1 diabetes mellitus ICD-10-CM N52 Male erectile dysfunction | 151,572 | 71 |
| **Group 2A -** The terms in this group occurred between Jan 01, 2019 and Sep 30, 2024. **Must Have:** ATC A10BH Dipeptidyl peptidase 4 (DPP-4) inhibitors [≥ 18 years]  **Group 2B -** Any instance of Group 2B occurred within 1 day and 1 year before the first instance of Group 2A. **Must Have:** Visit **Cannot Have:** ATC A10BH Dipeptidyl peptidase 4 (DPP-4) inhibitors | 114,997 | 70 |
| Results did not return | 337 | 70 |
|  | **114,660** | **70** |
|  | Patients | HCOs |

**Supplement Table 6.** Baseline characteristics of patients between GLP-1RA and. DPP4i groups using electronic health record data from TriNetX U.S. Collaborative Network (Jan 1, 2019 to Sep 30, 2024)

|  | **Before 1:1 PSM** | | | **After 1:1 PSM** | | |
| --- | --- | --- | --- | --- | --- | --- |
| **Characteristics** | GLP-1RA  (n=224,022) | DPP4i  (n=114,660) | SMD | GLP-1RA  (n=98,131) | DPP4i  (n=98,131 | SMD |
| Age at Index | 57.78(12.64) | 63.90(12.44) | 0.488 | 62.32 (11.77) | 62.38(12.28) | 0.006 |
| **Ethnicity** | | | | | | |
| Hispanic or Latino | 8.30% | 9.06% | 0.027 | 9.05% | 8.85% | 0.007 |
| Not Hispanic or Latino | 72.85% | 73.96% | 0.025 | 73.16% | 73.28% | 0.003 |
| Unknown Ethnicity | 18.85% | 16.98% | 0.049 | 17.80% | 17.87% | 0.002 |
| **Race** | | | | | | |
| White | 71.16% | 64.81% | 0.136 | 66.92% | 67.30% | 0.008 |
| Black or African American | 13.50% | 14.26% | 0.022 | 13.98% | 13.98% | 0.000 |
| Asian | 3.92% | 7.80% | 0.166 | 6.01% | 5.85% | 0.007 |
| Native Hawaiian or Other Pacific Islander | 0.88% | 0.86% | 0.003 | 0.86% | 0.85% | 0.001 |
| American Indian or Alaska Native | 0.63% | 0.59% | 0.005 | 0.63% | 0.62% | 0.002 |
| Unknown Race | 5.58% | 6.34% | 0.032 | 6.39% | 6.29% | 0.004 |
| Other Race | 4.32% | 5.34% | 0.048 | 5.21% | 5.11% | 0.005 |
| **Health care utilization** | | | | | | |
| Emergency Department Services | 50.56% | 45.26% | 0.106 | 46.10% | 45.82% | 0.005 |
| Preventive Medicine Services | 28.46% | 30.74% | 0.050 | 28.07% | 28.19% | 0.003 |
| Hospital Inpatient and Observation Care Services | 10.79% | 5.93% | 0.176 | 6.65% | 6.65% | 0.000 |
| Office or Other Outpatient Services | 0.60% | 0.11% | 0.082 | 0.18% | 0.13% | 0.013 |
| **Comorbidites** | | | | | | |
| Diabetic neuropathy | 58.99% | 60.03% | 0.021 | 58.82% | 58.46% | 0.008 |
| Diabetic nephropathy | 10.85% | 9.85% | 0.033 | 9.58% | 9.62% | 0.001 |
| Diabetes circulatory complications | 10.62% | 14.99% | 0.131 | 12.79% | 12.74% | 0.001 |
| Diabetic retinopathy | 11.09% | 11.28% | 0.006 | 11.05% | 11.02% | 0.001 |
| Other diabetes complications | 5.80% | 6.71% | 0.038 | 6.07% | 6.06% | 0.000 |
| Hypertension | 3.94% | 3.78% | 0.008 | 3.85% | 3.82% | 0.001 |
| Lipid disorders | 56.18% | 56.66% | 0.010 | 55.30% | 55.22% | 0.002 |
| Overweight and obesity | 32.35% | 18.89% | 0.312 | 21.03% | 20.94% | 0.002 |
| Cerebrovascular diseases | 5.08% | 9.04% | 0.155 | 7.27% | 7.16% | 0.004 |
| Ischemic heart diseases | 18.45% | 23.46% | 0.123 | 21.18% | 21.31% | 0.003 |
| Heart failure | 8.88% | 11.38% | 0.083 | 9.78% | 9.87% | 0.003 |
| Atrial fibrillation and flutter | 8.04% | 10.88% | 0.097 | 9.44% | 9.54% | 0.003 |
| Chronic kidney disease | 10.45% | 16.39% | 0.175 | 13.69% | 13.69% | 0.000 |
| Disorders of gallbladder, biliary tract and pancreas | 59.61% | 59.82% | 0.004 | 58.16% | 58.10% | 0.001 |
| Chronic lower respiratory diseases | 32.99% | 32.21% | 0.017 | 31.35% | 31.13% | 0.005 |
| Sleep disorders | 23.39% | 14.93% | 0.216 | 16.07% | 16.01% | 0.002 |
| Diseases of esophagus, stomach and duodenum | 15.91% | 17.19% | 0.034 | 15.95% | 15.96% | 0.000 |
| Neoplasms | 12.48% | 14.91% | 0.071 | 13.60% | 13.64% | 0.001 |
| COVID-19 | 9.87% | 16.20% | 0.189 | 12.87% | 12.76% | 0.003 |
| Substance use disorders | 10.73% | 12.48% | 0.055 | 11.43% | 11.54% | 0.003 |
| Glaucoma | 11.43% | 12.24% | 0.025 | 11.29% | 11.30% | 0.000 |
| Osteoarthritis | 10.88% | 10.74% | 0.004 | 10.35% | 10.47% | 0.004 |
| Mood disorders | 11.29% | 9.60% | 0.055 | 9.40% | 9.47% | 0.002 |
| Anxiety | 11.52% | 9.34% | 0.071 | 9.32% | 9.29% | 0.001 |
| Diseases of liver | 7.50% | 6.78% | 0.028 | 6.60% | 6.51% | 0.004 |
| Thyroiditis | 71.09% | 72.39% | 0.226 | 69.90% | 70.26% | 0.017 |
| Malnutrition | 68.21% | 66.85% | 0.029 | 65.83% | 65.53% | 0.006 |
| Other nutritional deficiencies | 0.36% | 0.24% | 0.022 | 0.25% | 0.25% | 0.000 |
| **Medications** | | | | | | |
| Metformin | 43.40% | 34.46% | 0.184 | 36.07% | 36% | 0.002 |
| Insulins and analogues | 21.01% | 26.21% | 0.123 | 23.73% | 23.50% | 0.005 |
| Sulfonylureas | 14.07% | 17.12% | 0.084 | 16.53% | 16.34% | 0.005 |
| Sodium-glucose co-transporter 2 inhibitors | 15.75% | 22.34% | 0.168 | 19.13% | 18.92% | 0.005 |
| Thiazolidinediones | 3.00% | 2.79% | 0.013 | 2.90% | 2.89% | 0.001 |
| Agents acting on the renin-angiotensin system | 27.03% | 30.83% | 0.084 | 28.41% | 28.56% | 0.003 |
| Beta blocking agents | 7.53% | 11.61% | 0.139 | 9.42% | 9.48% | 0.002 |
| Calcium channel blockers | 26.92% | 25.21% | 0.039 | 24.24% | 24.41% | 0.004 |
| Diuretics | 16.23% | 19.78% | 0.092 | 17.70% | 17.56% | 0.004 |
| Antihypertensives | 2.31% | 2.75% | 0.028 | 2.59% | 2.55% | 0.003 |
| Lipid modifying agents | 20.25% | 21.29% | 0.026 | 19.96% | 19.97% | 0.000 |
| Antiinflammatory and antirheumatic products, non-steroids | 48.53% | 46.68% | 0.037 | 45.77% | 45.80% | 0.001 |
| Corticosteroids for systemic use | 43.45% | 37.90% | 0.113 | 38.00% | 38.16% | 0.003 |
| Opioids | 26.30% | 34.85% | 0.186 | 30.80% | 30.86% | 0.001 |
| Antidepressants | 25.65% | 26.52% | 0.020 | 25.04% | 24.93% | 0.003 |
| Antipsychotics | 19.50% | 17.66% | 0.047 | 17.87% | 17.67% | 0.005 |
| Antiobesity medications | 17.39% | 19.30% | 0.049 | 17.72% | 17.78% | 0.002 |
| Anti-dementia drugs | 18.20% | 14.90% | 0.089 | 14.83% | 14.90% | 0.002 |
| Antithrombotic agents | 6.35% | 8.50% | 0.082 | 7.28% | 7.22% | 0.002 |
| Proton pump inhibitors | 0.55% | 1.45% | 0.091 | 0.96% | 0.92% | 0.004 |
| **Lab and vital signs^a^** | | | | | | |
| Body mass index |  |  |  |  |  |  |
| < 24.9 kg/m2 | 4.07% | 12.71% | 0.315 | 7.86% | 7.69% | 0.007 |
| 25-29.9 kg/m2 | 14.33% | 24.97% | 0.270 | 21.82% | 21.61% | 0.005 |
| 30-34.9 kg/m2 | 22.50% | 22.00% | 0.012 | 23.13% | 23.09% | 0.001 |
| 35-39.9 kg/m2 | 19.44% | 11.99% | 0.206 | 13.64% | 13.62% | 0.001 |
| ≥ 40 kg/m2 | 19.36% | 7.48% | 0.354 | 9.03% | 8.68% | 0.012 |
| HbA1c, ≥ 7 % | 48.88% | 46.55% | 0.047 | 47.45% | 46.74% | 0.014 |
| Systolic blood pressure, ≥ 130 mmHg | 56.05% | 52.85% | 0.064 | 51.95% | 51.86% | 0.002 |
| Diastolic blood pressure, ≥ 90 mmHg | 26.24% | 21.73% | 0.106 | 21.91% | 21.74% | 0.004 |

^a^The percentages of missing values were 32.56% for systolic blood pressure, 32.57% for diastolic blood pressure, 36.14% for body mass index, and 37.99% for HbA1c. Because imputation was not available in TriNetX, these variables were categorized using available values and included as categorical covariates in the analyses.

GLP-1RA, glucagon-like peptide-1 receptor agonists; DPP4i, dipeptidyl peptidase-4 inhibitors.

PSM, propensity score matching; SMD, standardized mean difference; HbA1c, glycated hemoglobin.

**Supplement text 1.** External Validation Using TriNetX Research Network

We conducted an external validation analysis using the TriNetX U.S. Collaborative Network, a federal, real-world research platform comprising de-identified electronic health record (EHR) data from multiple healthcare organizations. TriNetX provides access to structured clinical data, including demographics, diagnoses, procedures, medications, and laboratory measurements, and applies a standardized analytic framework across participating sites.

Using TriNetX U.S. Collaborative Network between January 2019 and September 2024, we included adults (**≥** 18 years) with type 2 diabetes (T2D) who newly initiated a glucagon-like peptide-1 receptor agonist (GLP-1RA) or dipeptidyl peptidase-4 inhibitors (DPP4i) during the study period. New use was defined as no prior record of the index medication class within the preceding 12 months. Patients with a documented diagnosis of erectile dysfunction (ED) prior to cohort entry were excluded to ensure incident outcome assessment. We also excluded patients with a diagnosis of type 1 diabetes and end-stage kidney disease or dialysis.

Similar to the primary analysis, the primary outcome, incident ED, was identified using ICD-10 diagnosis codes recorded after treatment initiation. Patients were followed from cohort entry until the first occurrence of ED, death, or study end, whichever occurred first.

Time-to-event analyses were conducted using Cox proportional hazards models as implemented by TriNetX, with hazard ratios (HRs) and 95% confidence intervals (CIs) reported. Baseline covariates available within TriNetX—including age, sex, comorbidities, diabetes-related complications, and concurrent medications—were balanced between exposure groups using propensity score matching implemented within the TriNetX analytic environment (The list of baseline covariates is present in **Table S6**). Propensity scores were estimated using logistic regression, and 1:1 nearest-neighbor matching with a caliper of 0.1 of the pooled standard deviation of the logit of the propensity score was applied, consistent with TriNetX default settings.

Because TriNetX imposes platform-specific constraints, certain analytic elements, such as weighting strategies and negative control outcome calibration, could not be implemented identically to the primary analysis. Accordingly, the TriNetX analysis was intended as an external, platform-based validation to assess the consistency and robustness of the primary findings rather than as a direct replication of the full target trial emulation framework.

**Supplement Figure 1.** The flowchart of patient selection using Penn Medicine electronic health record (EHR) data. GLP-1RA: glucagon-like peptide-1 receptor agonist; DPP4i, dipeptidyl peptidase-4 inhibitor.

1,923 patients were excluded:

- 1247 Prior use of DPP4i
- 676 prior use of GLP-1RA

22,578 adult men (aged > 18 years) using GLP-1RA or DPP4i between Jan 1, 2019 and Sep 30 2024 within Penn Medicine EHR data

12,355 patients using GLP-1RA or DPP4i

10,219 patients were excluded:

- 8,513 without a diagnosis of type 2 diabetes
- 250 with a diagnosis of type 1 diabetes
- 981 with a prior diagnosis of erectile dysfunction
- 475 with a diagnosis of end-stage renal disease or dialysis
- 4 with incorrect date

10,432 patients initiating GLP-1RA or DPP4i

- 4,864 GLP-1RA initiators
- 5,568 DPP4i initiators

**Supplement Figure 2.** Love plot of the absolute standardized mean difference (SMD) for baseline covariates before and after stabilized inverse probability of treatment weighting (sIPTW)


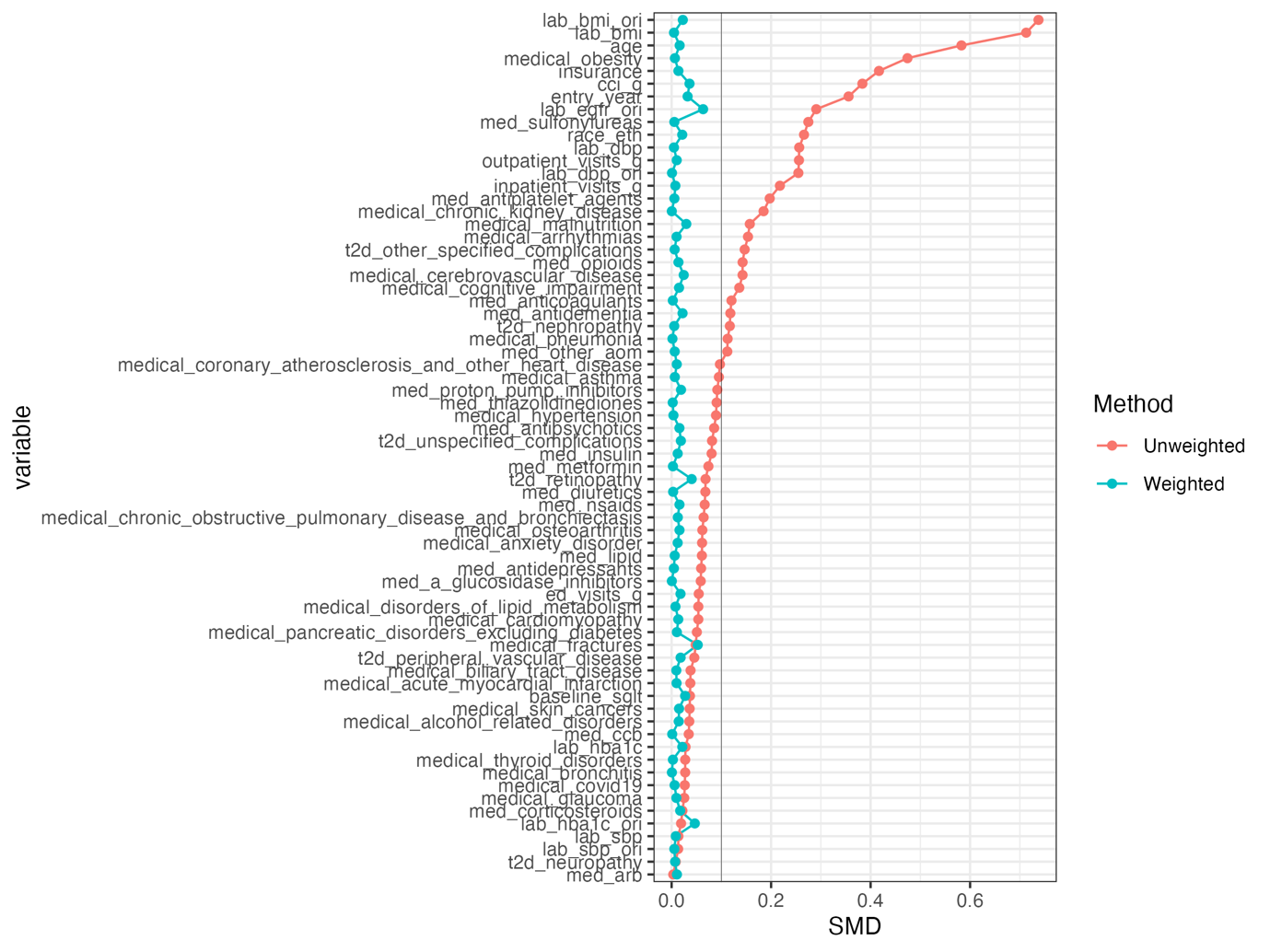


**Supplement Figure 3.** Empirical null distribution of negative control outcomes (NCOs). The plot displays hazard ratios and their corresponding standard errors for each NCO. To assess residual systematic error, we first derived the empirical null distribution of NCO using 1:1 stabilized inverse probability of treatment weighting (sIPTW) adjusted Cox proportional hazards models applied in the primary analysis. This empirical null was then used to calibrate the effect estimates. Additional details on the empirical null estimation and calibration procedures are provided in Schuemie et al. (2014, 2018) ^1,2^. The expected absolute systematic error quantifies the magnitude of systematic bias, with lower values indicating less bias and a value of 0 indicating no systematic bias.


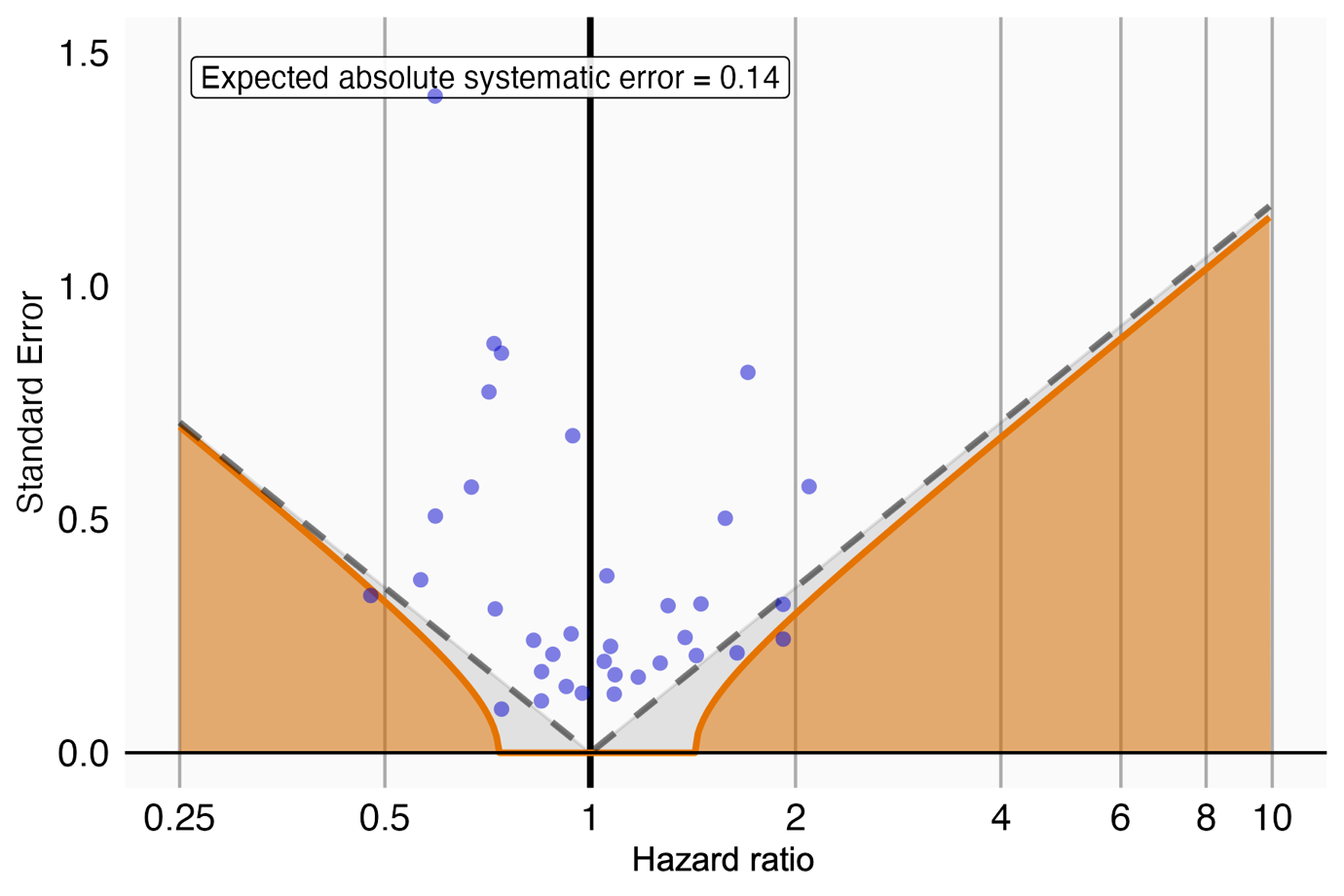


**References**

1. Schuemie MJ, Ryan PB, DuMouchel W, Suchard MA, Madigan D. Interpreting observational studies: why empirical calibration is needed to correct p-values. *Stat Med* 2014; **33**: 209–18.

2. Schuemie MJ, Hripcsak G, Ryan PB, Madigan D, Suchard MA. Empirical confidence interval calibration for population-level effect estimation studies in observational healthcare data. *Proc Natl Acad Sci U S A* 2018; **115**: 2571–7.
